# Genome-Wide Linkage Analysis of Global Gene Expression in Loin Muscle Tissue Identifies Candidate Genes in Pigs

**DOI:** 10.1371/journal.pone.0016766

**Published:** 2011-02-08

**Authors:** Juan Pedro Steibel, Ronald O. Bates, Guilherme J. M. Rosa, Robert J. Tempelman, Valencia D. Rilington, Ashok Ragavendran, Nancy E. Raney, Antonio Marcos Ramos, Fernando F. Cardoso, David B. Edwards, Catherine W. Ernst

**Affiliations:** 1 Department of Animal Science, Michigan State University, East Lansing, Michigan, United States of America; 2 Department of Fisheries and Wildlife, Michigan State University, East Lansing, Michigan, United States of America; 3 Department of Animal Sciences, University of Wisconsin, Madison, United States of America; 4 Embrapa Southern Region Animal Husbandry, Rio Grande do Sul, Brazil; King's College London, United Kingdom

## Abstract

**Background:**

Nearly 6,000 QTL have been reported for 588 different traits in pigs, more than in any other livestock species. However, this effort has translated into only a few confirmed causative variants. A powerful strategy for revealing candidate genes involves expression QTL (eQTL) mapping, where the mRNA abundance of a set of transcripts is used as the response variable for a QTL scan.

**Methodology/Principal Findings:**

We utilized a whole genome expression microarray and an F_2_ pig resource population to conduct a global eQTL analysis in loin muscle tissue, and compared results to previously inferred phenotypic QTL (pQTL) from the same experimental cross. We found 62 unique eQTL (FDR <10%) and identified 3 gene networks enriched with genes subject to genetic control involved in lipid metabolism, DNA replication, and cell cycle regulation. We observed strong evidence of local regulation (40 out of 59 eQTL with known genomic position) and compared these eQTL to pQTL to help identify potential candidate genes. Among the interesting associations, we found *aldo-keto reductase 7A2* (*AKR7A2*) and *thioredoxin domain containing 12* (*TXNDC12*) eQTL that are part of a network associated with lipid metabolism and in turn overlap with pQTL regions for marbling, % intramuscular fat (% fat) and loin muscle area on *Sus scrofa* (SSC) chromosome 6. Additionally, we report 13 genomic regions with overlapping eQTL and pQTL involving 14 local eQTL.

**Conclusions/Significance:**

Results of this analysis provide novel candidate genes for important complex pig phenotypes.

## Introduction

Integration of transcriptional profiling with genotyping data in segregating populations allows linkage mapping of expression quantitative trait loci (eQTL) [1], [2]. Over the last decade, such studies have been conducted in human cell lines and model organisms [3] and in plant species [2], [4], but in livestock species global eQTL experiments are still sorely lacking. In particular, genetical genomics studies in livestock have concentrated on experimental design and modeling issues [5], [6], [7], [8], on the analysis of selected transcripts [9] or on the comparative transcriptional profiling of genetically diverging lines [6], [10], and only recently has a global analysis been published for pigs [11].

The implementation of eQTL mapping has the potential to uncover gene networks and the genetic control of gene activity, as well as shed light on the genetic architecture of phenotypic variation, through integration with phenotypic QTL (pQTL) results [12].

To date, 5,732 pig QTL have been reported to the PigQTLdb database (http://www.animalgenome.org/QTLdb/) [13] for a total of 588 traits, but a very small proportion of these have materialized into causative variation associated with known genes [14].

The use of eQTL analysis has been demonstrated as a promising tool for narrowing the gap between detected pQTL regions and confirmed causative variants for the pig species [14]. Additionally, eQTL mapping can be used to reconstruct regulatory networks involving endpoint traits and expression traits, resulting in information useful for selection decisions [12]. For example, previous work has detected gene expression traits associated with pQTL for drip loss, an important pork quality trait [9], [15]. However, results were derived from a series of pre-selected transcripts based on phenotypic correlations with a trait of interest. This group has recently performed an eQTL analysis for *longissimus dorsi* transcripts and their association with meat quality traits [11], and they have identified over 9,000 eQTL at a suggestive significance threshold of LOD >2.

In this paper, we present a genome-wide linkage analysis of global gene expression using an F_2_ intercross of two pig breeds (Duroc and Pietrain). We used a sub-sample [7] from a large resource population that we created for pQTL mapping [16], [17]. We tested the expression of almost 20,000 transcripts on a recently developed microarray for linkage across the pig genome. Furthermore, we compared existing pQTL regions with local eQTL to identify candidate genes for traits of interest. We also performed gene set analysis to uncover regulatory networks subject to genetic control.

## Results

### Physical location of oligonucleotides

**Figure 1:**
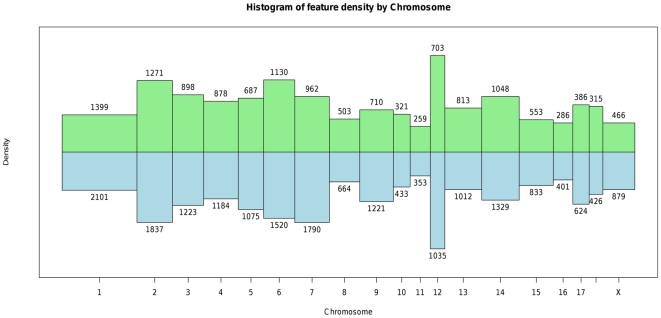
Histogram of oligonucleotide and gene densities across chromosomes. Green bars pointing up represent the distribution of oligonucleotides on the microarray. Blue bars pointing down represent the distribution of genes in the pig genome assembly (Build 9, www.ensembl.org). The bar width is proportional to chromosome length in base pairs, the height of the bar is proportional to the feature density, and the area of the bar is proportional to the feature count. Counts next to each bar represent oligonucleotide (green bars) and gene counts (blue bars).

To evaluate gene expression we used the swine protein-annotated oligonucleotide microarray (Pigoligoarray) [18]. This microarray includes 20,400 unique 70-mer oligonucleotides designed from contigs developed by comparison of expressed sequence tags to phylogenetically defined vertebrate proteins. Our previous publication [18] included comparative annotation for this array, but determining the physical positions of the oligonucleotides in the pig genome was not possible at that time. Determination of local and distant regulatory variation requires knowledge of the physical positions of probes on the expression profiling platform. We aligned 20,400 oligonucleotides from the Pigoligoarray [18] with the pig genome (Build 9; www.ensembl.org). The array included 19,980 non-control probes along with negative and mismatch probes, and we determined the positions for 13,611 oligonucleotides. Therefore, approximately one third of the probes could not be aligned to the current pig genome assembly. A list of these oligonucleotides and their positions is available in the Supporting Information (Table S1). The number of oligonucleotides per chromosome ranged between 286 for *Sus scrofa* (SSC) chromosome 16 to 1,399 for SSC1 (Figure 1). SSC12 presented the highest density of oligonucleotides per megabase, whereas SSC11, 16 and X had the lowest densities. We compared the physical distribution of oligonucleotides with the distribution of automatically annotated genes along the pig genome (Figure 1). Coverage was uniform across chromosomes as shown in Figure 1 where the relative number of genes and oligonucleotides for each chromosome was similar.

### Significance tests and putative eQTL

**Figure 2:**
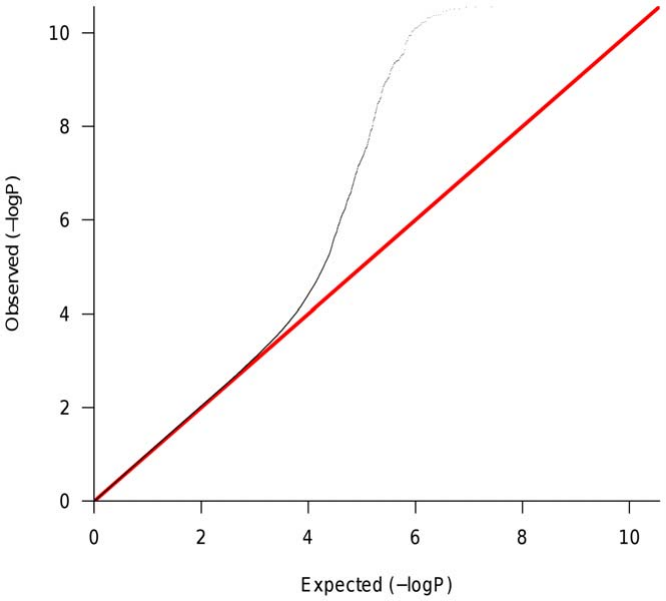
Quantile-quantile plot of p-values. Each point represents the p-value (log-scale) from a test. Expected values are plotted on the horizontal axis and observed values are plotted on the vertical axis. The expected distribution under the null hypothesis is represented by the diagonal red line. An excess of small p-values is observed compared to the null model represented by the red line.

**Figure 3:**
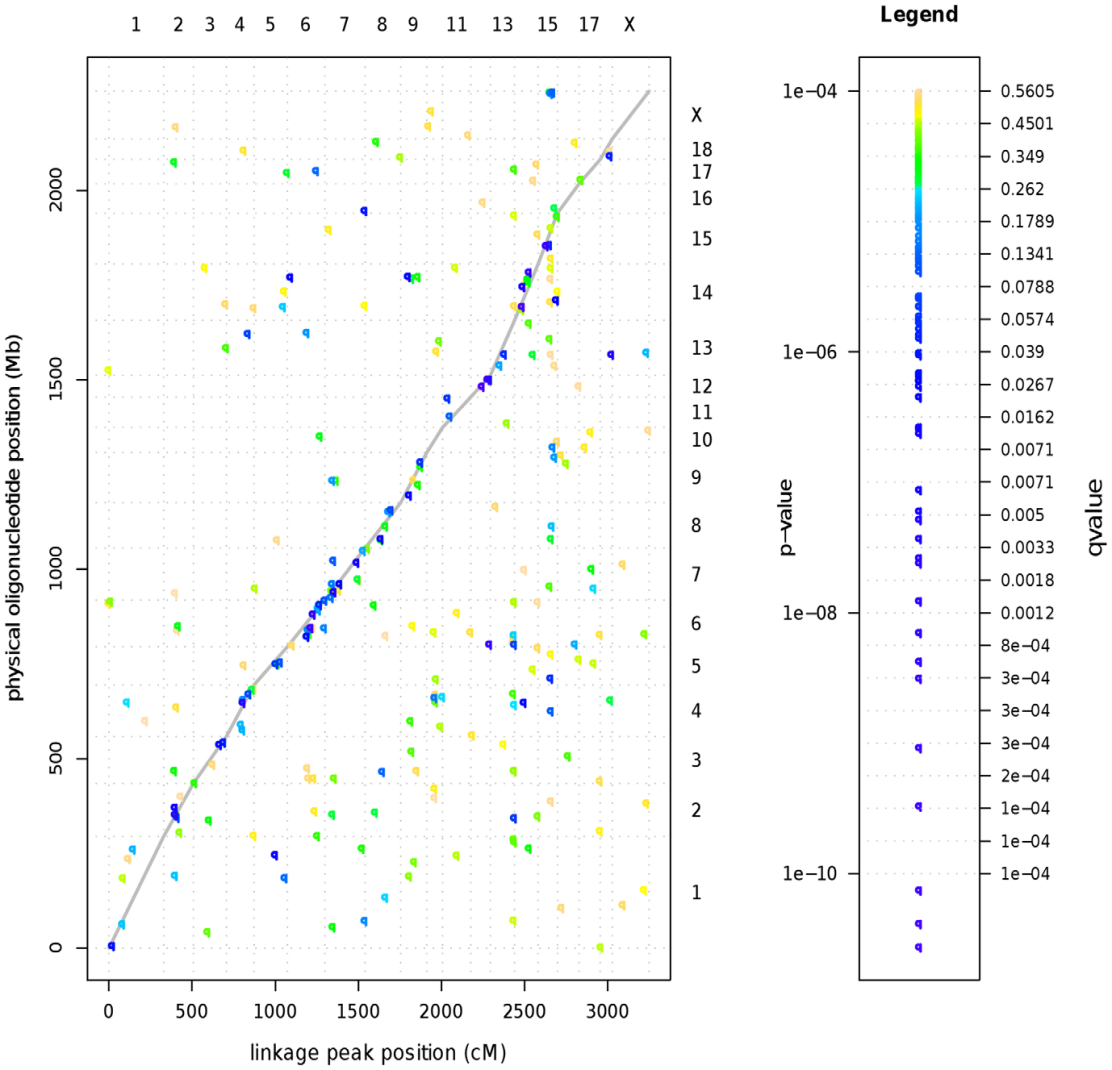
Global plot of physical position of oligonucleotide probe versus linkage position of eQTL across the pig genome. Points along the gray curve represent local eQTL (most likely cis-acting), while points off the line represent trans-acting eQTL. Colors represent increasing significance from yellow to green to blue to indigo.

The datasets for analysis included gene expression data (normalized log-intensity) for each transcript determined from *longissimus dorsi* muscle (loin muscle) tissue for each F_2_ animal, and genotype and phenotype information for a three generation pig pedigree (F_0_, F_1_ and F_2_ individuals). Genotype information was used to derive breed of origin probabilities across the genome of F_2_ animals at each marker and at 11 equidistant inter-marker positions yielding 1,279 putative QTL positions. We subsequently fit linear mixed models to each expression trait (20,400) and putative QTL position. A nominal p-value was used to test the null hypothesis of no eQTL at each position and expression trait combination. Testing for 1,279 putative QTL positions in almost 20,000 expression traits produced over 26 million p-values that required multiple test correction. Inspection of the quantile-quantile plots of p-values (Figure 2) revealed an excess of smaller p-values compared to what was expected under the null hypothesis. Using a p-value cutoff of P<0.0001, a total of 397 putative eQTL peaks were inferred and 253 of those were associated with oligonucleotides with known physical position. Notably, local (putatively cis-acting) eQTL had in general smaller p-values compared to trans-acting eQTL. The global pattern is represented in Figure 3.

**Figure 4:**
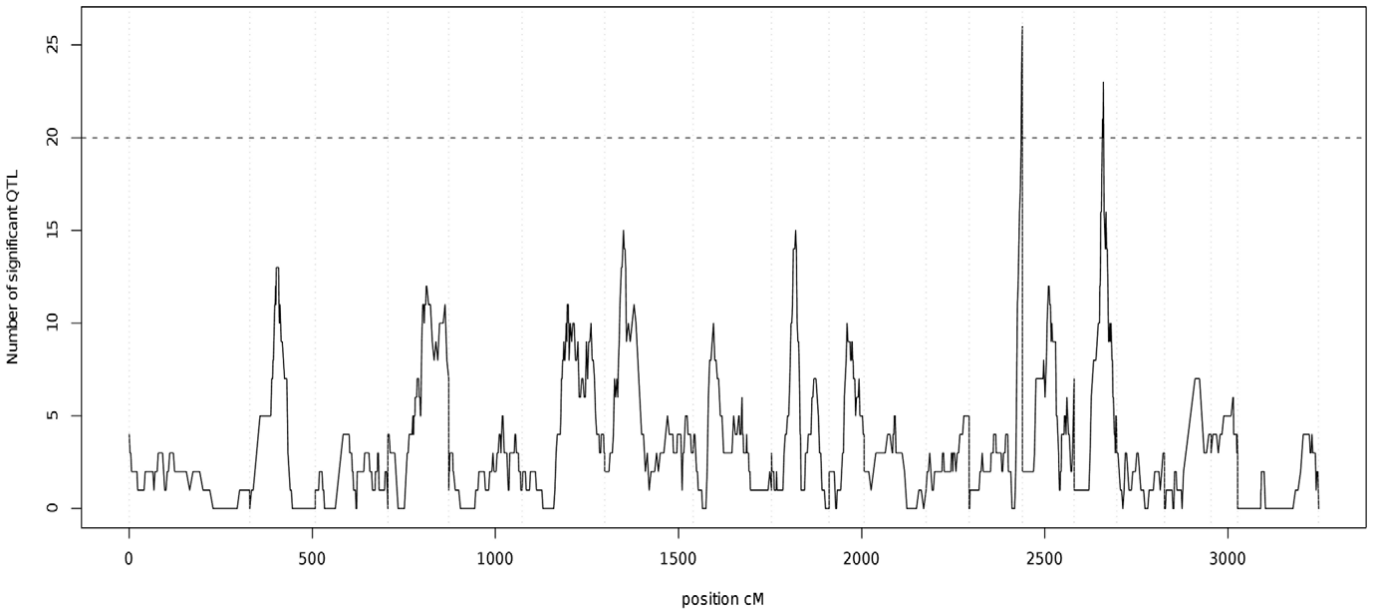
Number of putative eQTL per genomic position (p<0.0001). Vertical dotted lines represent the chromosome limits in the linkage map. The horizontal line indicates the expected number of significant eQTL under the null hypothesis (of no linkage). The number of putative eQTL rises above the threshold in only two hotspots. This indicates that despite finding strong evidence of eQTL, our study does not show evidence of the presence of hotspots.

For individual gene analyses, a more stringent significance threshold (P<0.0000035, FDR<10%) was used. This produced a total of 978 significant tests, corresponding to 62 unique linkage peaks comprising 59 genes with comparative human gene annotation (Tables 1 and 2). The positional analysis of these oligonucleotides indicated that 40 of these 59 eQTL were located on the same chromosome as the physical location of the oligonucleotide (local eQTL). Very limited evidence of hotspots of trans-regulation was found on only SSC13 and SSC15 after correction for multiple tests (Figure 4). At a nominal p-value threshold of P<0.0001, we would expect 20 false positives at any putative QTL position. The hotspot on SSC13 includes 27 eQTL and the hotspot on SSC15 includes 23 significant eQTL. We consider that these putative hotspots are actually not significant once we correct for multiple tests; a confirmation would require a computationally prohibitive permutation analysis [19] and consequently we do not pursue further study of trans regulation in this paper.

**Table 1:** Details of eQTL detected on Chromosomes 1–7.

Linkage peak							Annotation		Physical Position			
SSC	cM	Flanking markers		h^2^	Over^a^	p-value	OligoID	HGNC	SSC	Base pair	Method^b^	Local
1	21.2	SW1514	SW1515	4.7%	Du	7.00E-07	19030:2021_CL1Contig1:f	DYNLT1	1	9.6M	BLAT	YES
1	70.6	S0008	S0331	1.5%	Du	1.30E-07	35366:45360_37797494:f	UNKNW				
2	69.3	SW240	S0170	10.9%	Du	2.61E-07	11910:1243_CL4Contig1:f	CKMT2	2	79.1M	BLAT	YES
2	70.5	S0170	S0170	8.9%	Du	2.45E-08	12040:12202_CL2Contig7:r	CYP4F2	2	60.4M	BLAT	YES
2	72.6	S0170	SW1026	5.6%	Pie	6.10E-08	12039:12202_CL2Contig2:r	CYP4F3	2	60.4M	BLAT	YES
2	88.9	SW1026	S0370	5.7%	Du	1.08E-07	12429:2139_CL1Contig1:r	RFXANK	2	61.7M–63.6M	Comp	YES
3	158.4	SW2408	SW2408	9.2%	Du	6.62E-07	NM_213966.1	LCTHIO	3	105.2M	BLAT	YES
3	182.1	SW1327	SW2532	5.1%	Pie	9.99E-07	13665:2306_9018750:f	KCNS3	3	111.3M	BLAT	YES
4	76.4	SW2454	SW2454	4.0%	Du	1.27E-06	9659:11373_CL1Contig2:f	S100A1	4	83.3M–105.2M	Comp	YES
4	92.6	S0107	S0214	8.9%	Pie	2.90E-08	2602:3589_CL1Contig1:f	ZFAND1	4	0.9M–83.3M	Comp	YES
4	100.7	S0107	S0214	10.4%	Pie	1.26E-08	20010:19865_CL1Contig1:f	DUSP12	4	92.5M	BLAT	YES
4	131.1	S0214	S0097	3.1%	Du	2.60E-06	31129:32045_49416036:f	GSTM5	13	112.8M	BLAT	NO
4	131.1	S0214	S0097	8.0%	Du	1.30E-06	30881:32045_CL1Contig1:r	GSTM4	13	113.0M	Comp	NO
4	136.1	S0214	S0097	11.9%	Pie	2.72E-06	33801:32045_59789022:f	GSTM1	4	115M	BLAT	YES
4	136.1	S0214	S0097	12.8%	Pie	2.95E-07	30820:32045_34160672:f	GSTM1	13	113.0M	Comp	NO
4	141.1	S0214	S0097	7.9%	Pie	1.13E-10	33036:32045_CL3Contig1:r	GSTM4	13	113.0M	Comp	NO
5	129.2	SW2	S0005	8.3%	Du	2.69E-07	12773:4749_CL1Contig1:f	ZBTB5	1	249.6M	BLAT	NO
5	132.1	SW2	S0005	16.9%	Du	2.26E-06	10816:45360_CL475Contig1:r	CLEC1A	5	58.5M	BLAT	YES
6	20.4	S0099	SW2406	4.1%	Du	5.55E-07	19460:24177_CL1Contig1:f	Gcsh^d^	14	116.6M	BLAT	NO
6	118.9	S0087	S0220	13.4%	Du	6.83E-07	4057:25577_CL11Contig1:r	ETV2	6	30.3M	BLAT	YES
6	142.1	SW122	SW1881	6.1%	Pie	7.59E-11	5683:11259_CL1Contig1:r	AKR7A2	6	53.3M	BLAT	YES
6	149.0	SW122	SW1881	9.3%	Du	2.85E-10	31282:45360_CL420Contig1:f	ZNF24	4	115.0M	Comp	NO
6	155.8	SW122	SW1881	5.8%	Du	7.17E-09	9232:7693_25014272:r	SSX2IP	6	89.1M	BLAT	YES
6	194.4	SW1881	SW322	4.1%	Du	1.53E-06	9344:7136_CL1Contig1:r	TXNDC12	6	114.0M	BLAT	YES
6	220.8	SW322	SW2419	18.5%	Pie	2.74E-11	15007:3644_CL1Contig1:r	TMEM69	6	88.8M–122.2M	Comp	YES
7	55.4	S0064	SW1369	8.2%	Du	3.76E-08	34740:1138_CL1Contig12:mm2	HLA-A				
7	90.4	SW1369	SW859	13.6%	Pie	6.10E-07	32929:10370_CL1Contig2:r	MRPL14	7	45.6M	BLAT	YES
7	193.8	S0115	SWR773	7.8%	Pie	8.89E-08	688:4338_CL1Contig2:r	ERH	7	101.5M	BLAT	YES
7	241.5	SW764	SW764	6.7%	Du	3.54E-11	17750:32199_CL8Contig1:f	RAP2C				
7	241.5	SW764	SW764	1.8%	Du	9.90E-07	17698:46145_21549326:f	CDH12	16	9.4M	BLAT	NO

a Breed of origin of over-expressed allele: Duroc (Du) or Pietrain (Pie).

b Method used to determine oligonucleotide physical position: BLAST-Like Alignment Tool alignment of 70-mer (BLAT) or Comparative mapping of putative human gene onto pig genome sequence (Comp).

c Local QTL is called when oligo is on same chromosome as eQTL peak.

d Comparative mouse annotation available for this oligo; mouse genome symbol indicated instead of HGNC.

**Table 2:** Details of eQTL detected on Chromosomes 8 – 18 and X.

Linkage peak							Annotation		Physical Position			
SSC	cM	Flanking markers		h^2^	Ove^a^r	p-value	OligoID	HGNC	SSC	Base pair	Method^b^	Local
8	97.0	SWR1101	S0017	13.36%	Du	2.41E-07	11720:14916_CL3Contig1:f	LIMCH1	8	27.7M	BLAT	YES
8	157.9	SW1085	SW1085	6.15%	Pie	2.71E-06	14984:12042_CL2Contig1:r	METAP1	8	104M	BLAT	YES
9	46.9	SW983	SW911	7.19%	Pie	4.59E-07	8945:1411_CL2Contig2-B:r	SUFU	14	118.6M	NA	NO
9	53.3	SW911	SW911	9.29%	Du	6.10E-07	30905:27206_37855971:f	TYRL	9	22.7M	NA	YES
9	57.4	SW911	SW2401	5.76%	Du	1.61E-07	15326:18082_CL1Contig1:f	FOLH1	9	22.4M–22.9M	Comp	YES
9	121.3	SW989	SW2116	4.06%	Du	1.81E-06	3259:8737_CL1Contig1:r	MRPS14	9	110.0M	BLAT	YES
10	72.5	SW1041	SW920	4.44%	Pie	3.04E-06	35383:43456_59812215:r	UNKNW				
11	32.1	S0391	S0071	18.54%	Pie	1.91E-06	34282:31653_CL1Contig1:f	RNF17	11	79.6M	BLAT	YES
12	58.9	SW874	SW874	8.18%	Du	1.16E-07	2013:22635_CL1Contig1:f	PNPO	12	22.0M	Comp	YES
12	69.3	SW874	S0090	13.61%	Pie	2.78E-11	13785:2830_CL1Contig1:f	COIL	12	31.0M	BLAT	YES
12	103.3	S0090	SW2180	7.85%	Pie	3.35E-10	5144:16138_CL1Contig1:r	C17orf49	12	49.6M	BLAT	YES
12	114.1	S0090	SW2180	6.68%	Du	9.65E-07	5157:21325_CL2Contig2:f	RNF167	12	49.2M	BLAT	YES
12	114.1	S0090	SW2180	8.93%	Du	3.20E-09	32110:18719_CL1Contig1:f	EXOSC6	6	9.6M	BLAT	NO
13	85.4	S0068	SW398	1.83%	Du	1.70E-06	6679:12673_CL1Contig1:r	LSM3	13	58.6M	BLAT	YES
13	87.5	S0068	SW398	17.25%	Du	9.47E-10	153:9581_49327461:f	CDV3	13	61.0M	Comp	YES
13	143.5	SW2440	S0215	9.87%	Du	3.46E-06	195:8379_CL1Contig1:f	C21orf57				
13	145.4	S0215	S0215	7.25%	Pie	2.28E-06	12197:11213_CL1Contig1:f	TIMM44	2	51.3M	BLAT	NO
13	145.4	S0215	S0215	11.27%	Pie	2.64E-06	4546:6210_CL1Contig1:f	LDHD	6	9.0M	BLAT	NO
14	46.7	SW510	SW210	21.83%	Pie	4.20E-11	3659:36560_CL1Contig1:f	OAS2	14	39.1M	BLAT	YES
14	50.6	SW510	SW210	11.87%	Pie	5.27E-08	14615:7227_CL1Contig1:f	MMRN2	14	91.7M	BLAT	YES
14	56.3	SW210	SW210	5.24%	Pie	4.10E-07	14324:1651_CL2Contig1:r	GPX8	16	32M	BLAT	NO
14	58.2	SW210	SW886	6.48%	Du	4.60E-07	18899:4742_40469373:f	TOMM40L	4	92.7M	BLAT	NO
14	78.6	SW886	SW886	3.64%	Du	5.86E-07	8680:14598_CL1Contig1:r	AIFM2	14	76.0M	BLAT	YES
14	88.8	SW886	SW55	17.23%	Pie	9.39E-10	8339:13186_CL1Contig1:f	CASP7	14	129.9M	BLAT	YES
15	49.9	S0148	S0088	14.33%	Pie	4.31E-09	8463:8914_CL1Contig1:r	WRN	15	51.1M	BLAT	YES
15	66.0	S0148	S0088	12.87%	Pie	1.83E-06	15786:3609_CL2Contig1:f	OCA2	15		BLAT	YES
15	69.2	S0088	S0088	16.07%	Pie	1.38E-06	NM_214094.2	OCA2	15	53.6M	BLAT	YES
15	77.2	S0088	SW1683	4.52%	Pie	1.80E-06	11328:10595_CL1Contig1:r	CDK2	5	20.3M	BLAT	NO
15	111.1	SW1983	SW1119	1.27%	Du	6.16E-07	33697:14616_54517325:f	NID1	14	56.4M	BLAT	NO
18	59.8	SW1984	SW1984	5.40%	Du	1.30E-06	10346:26772_11504841:f	FAM180A	18	12.0M	BLAT	YES
18	69.5	SW1984	S0062	6.58%	Pie	2.67E-08	17270:2712_CL1Contig1:f	ZFYVE20	13	57.8M	BLAT	NO
18	71.7	SW1984	S0062	3.06%	Pie	3.63E-10	34616:32575_41143090:r	LRBA	8	67.3M	BLAT	NO

a Breed of origin of over-expressed allele: Duroc (Du) or Pietrain (Pie).

b Method used to determine oligonucleotide physical position: BLAST-Like Alignment Tool alignment of 70-mer (BLAT) or Comparative mapping of putative human gene onto pig genome sequence (Comp).

c Local QTL is called when oligo is on same chromosome as eQTL peak.

### Gene networks subject to genetic control

The p-values and fold-changes (relative expression of Duroc to Pietrain allele of origin) for significant eQTL were input into the Ingenuity Pathways Analysis software (Ingenuity Systems, Redwood City, CA, USA) for further data mining of pathways subject to genetic control. Three gene networks were enriched for differentially expressed genes between alleles of alternative breed origin (Table 3). This suggests that the corresponding eQTL genes influence loin muscle tissue accretion via common metabolic pathways. One network associated with lipid metabolism includes two members of the cytochrome P450 4F family of genes which are involved in the metabolism of long chain fatty acids, and these two genes were overexpressed in animals carrying the Duroc allele. This network also contains several genes including *aldo-keto reductase 7A2* (*AKR7A2*), *thioredoxin domain containing 12* (*TXNDC12*) and *translocase of inner micochondrial membrane 44* (*TIMM44*) that have functions related to oxidative stress and which were overexpressed in animals carrying the Pietrain allele. A second network associated with the cell cycle and lipid metabolism includes three members of the glutathione S-transferase mu family as well as *glutathione peroxidase 8* (*GPX8*), again showing an increase in expression of genes with functions related to oxidative stress in animals with the Pietrain allele. This network also includes two genes overexpressed in animals with the Duroc allele that function as transcriptional repressors (*zinc finger protein 24* (*ZNF24*) and *enhancer of rudimentary homolog* (*ERH*)). A third network associated with DNA replication, recombination and repair, the cell cycle, and cell death includes several genes that function in cell growth or the cell cycle which were all overexpressed in animals carrying the Pietrain allele (*cyclin-dependent kinase 2* (*CDK2*), *methionyl aminopeptidase 1* (*METAP1*), *suppressor of fused homolog* (*SUFU*), *synovial sarcoma X breakpoint 2 interacting protein* (*SSX2IP*), and *Werner syndrome RecQ helicase-like* (*WRN*)). These results support these genes and networks as promising candidates for sources of variation in growth, carcass merit and meat quality observed in this population.

**Table 3:** Description of three networks enriched for eQTL genes.

Associated terms	Gene Symbols^a^
Lipid Metabolism, Small Molecule Biochemistry, Post-Translational Modification	ACTN1, **AKR7A2,** CASP2, CASP4, **CASP7,** CCNE1, CTSD, CYP4F2, CYP4F3, DNAJA3, **DUSP12,** DYNLL2, DYNLT1, HNF4A, LSM3, MRPS14, PAWR, PNPO, PSMA1, **RAP2C,** RNF167, **S100A1,** SART3, SLC2A4, SOCS3, **TIMM44,** **TXNDC12,** UBE2M, **ZFYVE20**
Cell Cycle, Drug Metabolism, Lipid Metabolism	AIFM2, BYSL, **CASP7,** CBL, CCDC130, CCNE1, CEP70, **COIL,** ERH, FOLH1, **GCSH,** **GPX8,** **GSTM1,** **GSTM4,** **GSTM5,** IGSF21, **LRBA,** MAD1L1, MYO5B, MYO6, NID1, PINK1, PSME3, PTPRK, TGFB1, TP53, TSPYL2, **ZBTB5,** ZBTB16, ZNF24
DNA Replication, Recombination, and Repair, Cell Cycle, Cell Death	BCL2L12, CASP3, **CASP7,** **CDK2,** CENPC1, CKMT2, CTNNB1, CXCL12, DFFB, E2F4, EXOSC6, GAS2, HSH2D, KCNS3, LIMCH1, **METAP1,** MRPL14, MYC, **OAS2,** **RFXANK,** RNF17, **SSX2IP,** **SUFU,** **WRN**

a Gene symbols in **bold** denote genes with significant eQTL and overexpressed in animals carrying the Pietrain allele. Gene symbols that are underlined denote genes with significant eQTL and overexpressed in animals carrying the Duroc allele. Gene symbols in black denote genes in the network with no eQTL detected. Networks and associated terms were determined using the Igenuity Pathways Analysis software (Ingenuity Systems, Redwood City, CA, USA).

### Co-localization analysis

Analysis for pQTL was performed for over 60 growth, carcass merit and meat quality traits measured on the Michigan State University Duroc x Pietrain F_2_ resource population [16], [17]. Many of these traits were measured on (or were directly related to) the same tissue where mRNA abundance was measured (i.e., loin muscle). We subsequently performed a co-localization analysis between pQTL and eQTL, and significant pQTL traits observed in this analysis were related to muscle size and meat quality. Co-localization analysis of pQTL and eQTL traits revealed 62 overlapped eQTL/pQTL regions, significantly more than expected by chance. We grouped these into 13 common genomic regions (Table 4) on seven chromosomes. Thus, these loci are candidate genes for the pQTL. Four pQTL regions linked to muscle size traits overlap local eQTL for *DYNLT1* on chromosome 1, *TXNDC12* on SSC6, *MRLP14* on SSC7 and *WRN* on SSC15. Traits related to meat color have pQTL overlapping eQTL on SSC15 (*OCA2*) and SSC8 (*LIMCH1*, *METAP1*). Expression QTL regions on SSC6 (*AKR7A2*) and SSC12 (*PPNO*, *COIL*) overlap pQTL for intramuscular fat traits. Moisture content had three pQTL regions coincident with local eQTL (*PPNO*, *COIL* on SSC12, *ETV2* on SSC6 and *LIMCH1* on SSC8). Protein content had two pQTL regions associated with eQTL, one on SSC6 that also coincided with pQTL for muscle size (*TXNDC12*) and another on SSC15 (*CDK2*). A pQTL region for meat tenderness overlapped the eQTL for *WRN* (SSC15) that also coincided with pQTL for muscle size. Ultimate meat temperature had a pQTL coincident with an eQTL for *TMEM69* on SSC6. Finally, a single eQTL region for loin muscle off-flavor on SSC2 overlapped a local eQTL for *RFXANK*.

**Table 4:** Overlapping eQTL and pQTL regions.

SSC	cM^a^	eQTL (gene symbols)	pQTL	p-value^b^
1	8–26	DYNLT1	Loin muscle area	0.006
2	84–100	RFXANK	Meat off -flavor	0.004
6	106–124	ETV2	% Moisture	0.005
6	137–147	AKR7A2	Marbling, % Fat	<0.0001
6	180–199	TXNDC12	% Protein, Loin muscle area, Loin chop weight	0.006
6	216–226	TMEM69	Ultimate meat temperature	<0.0001
7	85–101	MRPL14	Carcass length, Loin muscle area	0.003
8	83–111	LIMCH1	b^*^, % Moisture	0.006
8	153–168	METAP1	L^*^	0.003
12	46–74	PNPO, COIL	% Fat, % Moisture	0.004
15	45–64	WRN	Loin muscle area, Tenderness	0.009
15	55–74	OCA2	L^*^, a^*^, Meat color	0.002
15	72–85	CDK2	% Protein	0.007

a Total length of the overlapped region in cM.

b Largest overlap p-value between pQTL and eQTL traits.

## Discussion

We performed a genome-wide linkage analysis of global gene expression in a segregating swine population. Previous studies of eQTL in pigs used a global expression array [9], but the analysis itself was restricted to a set of pre-selected genes based on correlations with a trait of interest [9], [15]. In contrast, we did not pre-screen probes on the microarray for differential expression or correlations, but instead performed a QTL scan for transcript abundances derived from each probe. In this way, we did not bias the eQTL discovery towards genes correlated with a particular trait at the expense of introducing multiple testing that when accounted for may result in less power. Recently, Ponsuksili et al. [11] performed QTL analysis of expression traits in *longissimus dorsi* muscle of a Duroc x Pietrain cross. They used a different significance criteria than we used in our study leading to over 9,000 putative eQTL. Among their findings, they identified local eQTL for *OCA2* and *AKR7A2* in similar chromosomal positions to local eQTL for these transcripts that we identified in our study.

Deriving eQTL profiles for each of 20,000 probes is a computationally prohibitive task using publicly available software for QTL mapping in crosses of outbred lines [20]. Consequently, we programmed a computationally tractable implementation of the line cross model [21], that can accommodate random and fixed effects as required by two-color microarray data [22]. All programs are available upon request from the authors.

The expression platform used was the Pigoligoarray [18], which allowed us to infer local (on the same chromosome) or distant eQTL for over 13,000 probes. Comparatively, an alternative expression platform from the Affymetrix company currently has less than 9,000 probesets mapped to the pig genome (www.ensembl.org; queried April 2010 using http://www.biomart.org/). The physical position of oligonucleotides on the Pigoligoarray was generated as part of this work and is made available as additional annotation (Table S1).

To set a threshold for declaring statistical significance, we used a stringent p-value cutoff of P<3.5×10^−6^. Due to the many simultaneous tests performed, this resulted in a FDR of 10%. Even with this significance level we found 62 eQTL, of which 40 had linkage peaks on the same chromosome where the oligonucleotide was physically located.

This analysis has revealed new gene targets for further validation as potential genes controlling variation in pig pQTL traits. Among the local (potentially cis-acting) eQTL genes, many have limited functional information reported for any species. However, for eQTL genes that have been studied, although they would not have been obvious functional candidates for the pQTL trait phenotypes, consideration of their known functions can reveal potentially new biological roles for these genes in pig skeletal muscle.

The genes encoding *aldo-keto reductase 7A2* (*AKR7A2*) and *thioredoxin domain containing 12* (*TXNDC12*) both have roles in oxidative stress and cellular detoxification. Our results revealed a significant *AKR7A2* eQTL coincident with pQTL for % intramuscular fat and marbling traits on SSC6. While *AKR7A2* has been shown to be ubiquitously expressed in numerous human tissues including skeletal muscle [23], no reports have examined *AKR7A2* in skeletal muscle under varying physiological states. However, Picklo et al. [24] reported *AKR7A2* expression to be elevated in cerebral cortexes of Alzheimer's disease patients, a disease associated with elevated aldehyde products. *TXNDC12* has also been shown to be expressed in many human tissues including skeletal muscle [25]. While no studies have considered the function of *TXNDC12* in skeletal muscle, its role as a thiol-disulfide oxidoreductase in other cell types has been demonstrated [26], [27], [28]. Our results indicate SSC6 pQTL for protein content, loin muscle area (LMA) and loin muscle chop weight coincident with an eQTL for *TXNDC12*.

An eQTL for *dynein light chain Tctex-type 1* (*DYNLT1*) coincident with a pQTL for LMA was observed on SSC1. *DYNLT1* is a component of the dynein complex which is part of the microtubule-based motile process within the cellular cytoskeleton [29]. Microtubules are not present in appreciable amounts in the cytoskeleton of skeletal muscle [30]. *DYNLT1* is most highly expressed in human immune system cells and testis, and is expressed at relatively lower levels in skeletal muscle (BioGPS; http://biogps.gnf.org/), although expression was detected in all tissues examined by Watanabe et al. [31] using northern blot analysis, with skeletal muscle exhibiting relatively high abundance compared to other tissues. Reports regarding the function of *DYNLT1* have focused on the role of this gene in mouse neuron development [32] and have included observations of direct interaction of this protein with specific neuronal Ca2+ channels in rats [33]. Further research will be needed to determine if this gene has a similar function in pig skeletal muscle tissue.

*Werner syndrome protein* (*WRN*) is a member of a family of RecQ helicases that are involved in maintenance of genome stability. *WRN* has roles in DNA replication and repair, transcription, and telomere maintenance, and defects in *WRN* cause Werner syndrome, an autosomal recessive disorder associated with premature aging [34]. Our results identified a *WRN* eQTL coincident with pQTL for LMA and tenderness on SSC15. While no studies have been reported regarding the role of *WRN* in skeletal muscle, the functions of this protein in cell growth and transcription support the potential effects on muscle size and meat quality phenotypes observed in this study and indicate that further study is warranted.

An eQTL for *cyclin-dependent kinase 2* (*CDK2*) coincident with a pQTL for protein content was observed on SSC15. *CDK2* is involved in control of the cell cycle and also has cell cycle independent functions including DNA damage repair [35]. In skeletal muscle, *CDK2* has been shown to be a part of the mechanism that tightly controls MyoD levels and subsequent myoblast cell cycle progression or exit into differentiation [36]. In addition, Thomas et al. (2000) demonstrated with in vitro studies that myostatin decreased levels and activity of *CDK2* in myoblasts and also altered expression of other cell cycle components which led to arrest of myoblasts in the G(1)-phase of the cell cycle. While no studies have considered the function of *CDK2* in postnatal muscle satellite cells or mature myofibers, further study is needed to evaluate the potential role of this gene in pig skeletal muscle growth and to confirm the association of this gene with skeletal muscle protein content.

The *oculocutaneous albinism II* (*OCA2*) gene has been studied extensively for its role in the mammalian pigmentary system [37]. Allelic variants of *OCA2* define human blue-brown eye color; *OCA2* also functions in melanin synthesis within melanocytes, and aberrant *OCA2* alleles cause type 2 oculocutaneous albinism in humans. Our results identified a significant *OCA2* eQTL coincident with a pQTL for both objective and subjective meat color phenotypes on SSC15. No previous studies have reported an association of this gene with muscle color. Additional research will be needed to confirm this association and to determine if the cellular mechanism involves tyrosine transport similar to the mechanism in melanocytes.

This whole genome linkage analysis of global gene expression provides insight into genes and gene networks subject to genetic control in a segregating pig population. Individual gene analyses allowed identification of candidate genes in previously mapped pQTL regions.

## Methods

### Animals and genotyping

The Michigan State University (MSU) pig resource population is an F_2_ cross originated from 4 F_0_ Duroc sires and 16 F_0_ Pietrain dams. The full pedigree includes a single large family of 20 F_0_, 56 F_1_ and 954 F_2_ animals. We have previously published details of this cross [17]. Briefly, from the F_1_ progeny, 50 females and 6 males were retained to produce the F_2_ generation. Pigs were weaned at 16 to 25 (mean 19.8) days of age and moved into nursery pens. All diets fed were MSU standard swine farm diets that met or exceeded National Research Council [38] requirements for all nutrients at each production stage. At 10 weeks of age, F_2_ pigs were moved into finishing facilities at the MSU Swine Teaching and Research Farm. Pigs had *ad libitum* access to feed and water.

Of the 954 total F_2_ animals for which growth, carcass and meat quality phenotypes were recorded, 510 F_2_ pigs along with the F_0_ and F_1_ pigs were genotyped for 124 microsatellite markers (3–9 makers per chromosome) in an initial genome scan [16], [17]. These 510 animals were sampled from 61 litters across all farrowing groups and represented all F_1_ sires with at least 100 grand progeny from each F_0_ sire. By using the three generation pedigree, the breed of origin probability for each putative QTL position can be obtained if the QTL is assumed fixed for alternative alleles in each breed.

### Ethics statement

Experimental procedures were approved by the All University Committee on Animal Use and Care at Michigan State University (AUF# 09/03-114-00).

### Phenotype collection

Details of growth, carcass and meat quality phenotypes collected on the MSU Duroc x Pietrain resource population have been reported previously [16], [17]. Briefly, live animal traits collected on the F_2_ pigs included body weight at birth, weaning, and 6, 10, 13, 16, 19 and 22 weeks of age. In addition, B-mode ultrasound (Pie Medical 200SLC, Classic Medical Supply Inc., Tequesta, FL) estimates of 10^th^-rib backfat (BF10), last-rib backfat (LRF), and loin muscle area (LMA) were recorded at 10, 13, 16, 19 and 22 weeks of age. The average daily gain from 10 to 22 weeks of age and the number of days to reach 105 kg were calculated from these body weight measures [17]. At each of these timepoints, measures of fat-free total lean (FFTOLN), total body fat tissue (TOFAT), empty body protein (EBPRO), and empty body lipid (EBLIPID) were calculated [17]. For slaughter, pigs were transported to one of two abattoirs. A total of 176 pigs were slaughtered at the federally inspected MSU Meat Laboratory (East Lansing, MI) to facilitate tissue collection for the current study, and the remaining pigs were slaughtered in a federally inspected plant in western Michigan (DeVries Meats, Coopersville, MI). Slaughter age was 165.8±9.2 d (112±9 kg live body weight). Carcass traits collected included hot carcass weight (HCW), and loin muscle pH and temperature at 45 min and 24 hour postmortem. Dressing percentage was calculated by dividing HCW by live slaughter weight. After overnight chilling, midline first-rib backfat, last-rib backfat, last-lumbar backfat, number of ribs, and carcass length measurements were recorded [16]. Weights of primal cuts of ham, closely trimmed loin, picnic shoulder, Boston shoulder, belly and spareribs were also recorded [16]. A section of loin from the 10^th^ rib to the last rib of the left side of the carcass was further evaluated for meat quality traits at MSU. Traits included scores for subjective color, marbling and firmness, objective color scores of CIE L* (lightness), a* (redness) and b* (yellowness) using a Minolta CR-310 colorimeter, drip loss, cook yield, and Warner Bratzler shear force [16]. Samples were also evaluated for proximate composition (moisture, fat and protein) and a trained sensory panel evaluated samples for juiciness, muscle fiber and overall tenderness, connective tissue, and off-flavor [16].

### Gene expression

We utilized a recently developed pig whole-genome 70-mer oligonucleotide microarray for this study. The swine protein-annotated oligonucleotide microarray, or Pigoligoarray, was evaluated by our group for use in pig gene expression studies and we reported on the utility of this microarray, as well as information regarding hybridization and analysis methods [18]. The Pigoligoarray includes 20,400 oligonucleotides. Probes were designed from contigs by comparison of pig expressed sequence tags (ESTs) to phyogenetically defined vertebrate proteins and were annotated using descriptions of homologous proteins (http://www.pigoligoarray.org). The microarray includes 60 negative control probes and six mismatch hybridization stringency control probes designed against each of 60 contigs with the highest EST count in the database. Further annotation of Pigoligoarray probes to include HUGO Gene Nomenclature Committee (HGNC) identities was reported by our group [18].

*Longissimus dorsi* (loin) muscle tissue was sampled from 176 F_2_ pigs slaughtered in the MSU Meat Laboratory. The 176 F_2_ pigs were selected from 44 litters (4 per litter). Within each litter, pigs were selected for transcriptional profiling using a selective phenotyping strategy which consisted of choosing the two extreme males and females within each litter for a trait of interest. For 24 litters loin muscle area was the selection criteria while for the other 20 litters, the extreme pigs were selected based on backfat depth. This selective profiling strategy has been previously described by our group [7]. Tissue samples were flash frozen in liquid nitrogen and stored at – 80°C. Total RNA from 1.0 g of each tissue sample was extracted using TRIzol reagent (Invitrogen Corp.) according to the manufacturer's instructions. RNA concentration and quality were determined with an RNA 6000 Pico LabChip® kit using an Agilent 2100 Bioanalyzer (Agilent Technologies, Inc.). Samples were paired for microarray hybridizations within sex and within litter such that extreme phenotype males and extreme phenotype females within a litter were paired on microarrays. In addition, dyes were balanced so that, within a litter, samples of high phenotype males and low phenotype females had the same dye, and each dye was used equally for each phenotype group across the experiment.

Sample preparation, microarray hybridization and processing were as previously described by our group [18]. Briefly, for each sample, 1 µg of total RNA was reverse transcribed with a T7 oligo(dT) primer using the Amino Allyl MessageAmp™ II aRNA Amplification Kit (Ambion Inc.) according to the manufacturer's instructions. After aRNA purification, 10 µg of aRNAs were labeled with N-hydroxysuccinate ester Cy3 or Cy5 dyes (GE Healthcare). The labeled aRNAs (2.5 µg) were purified, combined with Slide Hyb #1 solution (Ambion Inc.) and denatured at 70°C. Hybridizations were performed in sealed cassettes (ArrayIt, TeleChem International, Inc.) for 18 h at a humid 54°C. Following hybridization, slides were washed and rinsed as we have reported previously. Fluorescent images were detected by an Axon GenePix® 4000B scanner (Molecular Devices), and fluorescence intensity data were collected using GenePix® Pro 6 software (Molecular Devices) after spot alignment. The gene expression data were deposited in the NCBI Gene Expression Omnibus database (http://www.ncbi.nlm.nih.gov/geo/) [accession number GSE23351]. Median intensities were extracted and normalized using a within-print-tip lowess location normalization and an overall scale normalization [39] with no background correction. This normalization removed intensity dependent biases from each printing block in each slide. The resulting normalized data were expressed in the log_2_ scale.

### Statistical analysis

The following linear mixed model was fit to normalized log-intensity data on an oligonucleotide-by-oligonucleotide basis:
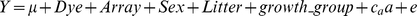
where *Y* is the normalized log-intensity, *Dye*, *Sex* and *growth_group* are fixed effects accounting for systematic variation, and *Array* and *Litter* are random effects. Growth group had four categories corresponding to the extremes for selection criteria: 1) High loin muscle area, 2) low loin muscle area, 3) high back fat and 4) low back fat. The additive eQTL coefficient *c_a_* was derived assuming that the parental breeds were fixed for alternative eQTL alleles [21]. A t-test for the additive eQTL effect *a* was performed at each of the 1,279 putative eQTL positions (at every marker and 11 inter-marker positions) for each expression trait and the p-values were corrected for multiple testing (q-value) across all traits and positions. A preliminary analysis considered P<0.0001 (FDR<56%) as significance threshold. Candidate eQTL analysis on individual genes used P<0.0000035 (FDR<10%).

### Physical localization of oligonucleotides relative to localization of eQTL

All oligonucleotides were aligned against the pig genome (Build 9; www.ensembl.org) using the BLAT [40] sequence alignment tool. Up to 3 mismatches were allowed and multiple alignments of the same oligonucleotide sequence were discarded as they indicated ambiguous positions due to problems with the genome assembly or with the oligonucleotide design. By comparing the position of each significant linkage peak to the physical position of the corresponding oligonucleotide, local and distant eQTL were declared. We declared local eQTL as those where the linkage peak and the oligonucleotide in question were on the same chromosome and distant eQTL as those where the eQTL and the oligonucleotide were on different chromosomes. All distant eQTL are trans-acting, whereas only a proportion of the local eQTL are cis-acting. While the local eQTL are putatively cis-acting, it is not possible to make definitive determinations from the current map resolution with the available genomic sequence and marker density.

### Gene networks subject to genetic control

Oligonucleotide annotation was obtained from our previous work [18]. The corresponding HGNC name and its associated QTL p-value and fold-change (relative expression of Duroc and Pietrain allele of origin) were input into the Ingenuity Pathways Analysis software (Ingenuity Systems, Redwood City, CA, USA) to test for enrichment of functional categories.

### Co-localization of eQTL and pQTL

A set of 67 phenotypic traits has been previously analyzed for QTL (pQTL) in this cross [16], [17]. For the current study, we repeated the pQTL analysis to account for a different mapping function and to compute multiple test p-value corrections (FDR). Standard QTL analysis approaches were applied as we have previously reported [16], [17]. Given a particular eQTL region, delimited by a 5 cM interval to each side of the peak, all overlapping pQTL regions were selected [4]. The probability of two intervals of this length (10 cM) overlapping in a 3,000 cM long genome (the length of our linkage map) is 
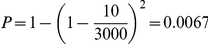
. We computed a p-value for observing (by chance) an overlap as long as the one observed in each eQTL/pQTL region (distance between center of intervals smaller or equal to the observed one) using this expression: 
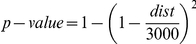
, where dist is the distance between two eQTL. Note that this formula does not depend on the length of the interval. The formulas were derived assuming uniform distribution of the center of the interval and verified using Monte-Carlo simulation.

## Supporting Information

Table S1 **Position of oligonucleotides in the pig genome.** Position is relative to the beginning of the chromosome (SSC). Pig Genome information corresponds to Build 9; accessible at: www.ensembl.org.
(XLS)
